# Evaluation of a Digital Protocol for Pre-Surgical Orthopedic Treatment of Cleft Lip and Palate in Newborn Patients: A Pilot Study

**DOI:** 10.3390/dj7040111

**Published:** 2019-12-09

**Authors:** Domenico Dalessandri, Ingrid Tonni, Laura Laffranchi, Marco Migliorati, Gaetano Isola, Stefano Bonetti, Luca Visconti, Corrado Paganelli

**Affiliations:** 1 School of Dentistry, Department of Medical and Surgical Specialties, Radiological Sciences, and Public Health, University of Brescia, Piazzale Spedali Civili 1, 25123 Brescia, Italy; ingrid.tonni@unibs.it (I.T.); laura.laffranchi@unibs.it (L.L.); s.bonetti63@gmail.com (S.B.); l.visco@libero.it (L.V.); corrado.paganelli@unibs.it (C.P.); 2 Department of Orthodontics, School of Dentistry, University of Genova, Largo Rossana Benzi 10, 16132 Genova, Italy; marco.migliorati@unige.it; 3 School of Dentistry, Department of General Surgery and Medical and Surgical Specialties, University of Catania, Via S. Sofia 78, 95123 Catania, Italy; gaetano.isola@unict.it

**Keywords:** cleft lip and palate, PSOT, molding plate, digital oral impression, gingivoperiosteoplasty

## Abstract

The aim of this study was to evaluate the accuracy, invasiveness and impact on clinical results of a digital oral impression protocol in the pre-surgical orthopedic treatment (PSOT) of newborn cleft lip and palate (CLP) patients undergoing primary alveolar surgical repair. Six patients were divided, according to impression technique used, into a digital (intraoral scanner (IOS)) and a non-digital (tray and putty (T&P)) group. Parents considered IOS impressions to be less invasive, compared to T&P impressions. The clinician that took all the impressions considered the IOS to be less stressful compared to the T&P method. In two T&P patients, the impression was repeated because some important anatomical details were missing, in one case due to patient regurgitation during the first attempt. No impression was repeated, and any adverse event was reported in the IOS group. There were no significant differences between these two protocols in pre-surgical alveolar gap reduction and surgical challenge. The study results indicate that this digital protocol can accelerate the production process of the passive molding plate with an instantaneous transmission of the digital impression to the dental lab, maintaining the same accuracy level and clinical outcomes of classical techniques and reducing the invasiveness of impression taking, avoiding any risk of impression material ingestion or inhalation.

## 1. Introduction

Without proper early management, a complete unilateral cleft lip and palate patient growth is characterized by a severe nasal deformity, an outward, non-cleft side, maxillary alveolar process rotation and a medial movement of the cleft side, smaller alveolar segment [1,2]. Primary alveolar surgical repair is therefore done early in the cleft lip and palate (CLP) patient life, frequently after pre-surgical orthopedic treatment (PSOT) with an acrylic intra-oral passive molding plate, allowing the patient to feed, separating the oral from the nasal cavity, and guiding the growth process of maxillary stumps—“alveolar molding”—in order to facilitate surgery and ameliorate the results achieved [3,4,5]. The alveolar molding is accomplished through serial adjustments, removing hard acrylic from the areas the segments are being directed toward and adding soft liner material to the regions where force must be applied to the segments to accomplish the movement. Weekly visits are required to modify the molding plate to guide the alveolar cleft segments into the desired position [6,7,8]. Sometimes, this plate has to be substituted with additional impressions, in order to follow patient growth [9,10,11].

A 2016 study demonstrated that the proposed benefits of earlier forms of presurgical orthopedics, e.g., improved feeding, growth guidance, development of palatal segments, minimization of treatment at a later age, and normalization of tongue position, resulting in better speech and a positive psychological effect on the parents, are unsubstantiated [12]. The current state of the art in molding techniques involves not only the alveolar processes but also the nose: correction of the nasal cartilage deformity, stretching of the nasal mucosal lining and achievement of nonsurgical columella elongation must be combined with the molding of the alveolar process before gingivoperiosteoplasty [13]. This is possible because of neonatal cartilage plasticity, due to high levels of circulating hyaluronic acid, that allow the correction of the nasal cartilage and soft tissue deformity [14]. Pre-surgical nasoalveolar molding results in long-term benefits to the patient and medical economics [15,16].

Intra-oral scanners (IOS) could replace the tray and putty (T&P) method of impressing patients [17,18,19]. They are constituted of a digital camera that consecutively acquires several pictures of the area to be impressed and subsequently transforms these two-dimensional images, token from different perspectives, into a single tridimensional reconstruction [20,21,22]. Older scanners required the use of an opaque powder spray, in order to cover the surfaces to be impressed before image acquisition, that is not necessary with the newer one, thus reducing patient discomfort and procedure complexity [23]. An IOS-based digital protocol could optimize the impression procedure because acquired data are instantly available to the dental lab for CAD (Computer Aided Design): only three minutes, on average, are needed to acquire one dental arch reconstruction [24,25]. IOS accuracy has improved in recent years and nowadays is comparable to traditional polyvinilsiloxane (PVS) materials [26,27].

The patient’s dental impression experience is quicker and more comfortable than traditional T&P methods and overall chair-time is reduced [28]. One operator is enough to obtain a very good quality impression, whereas, with traditional methods, the principal operator needed the help of a second operator in order to keep the dental arch to be impressed perfectly dry, and a third operator to mix impression materials and put them into impression trays [29,30]. Furthermore, there is no need to order and store impression materials of different viscosities that need to be used before their expiration date and must be manipulated with proper powder-free gloves [31]. The impression procedure’s safety and comfort are of crucial importance in CLP newborn patients and must be extensively considered when giving parents information, in order to obtain their informed consent [32].

The aim of this pilot study was to test the application of an IOS-based digital workflow protocol for orthopedic pre-surgical treatment of CLP newborn patients, evaluating impression and plate construction accuracy, invasiveness, and impact on clinical results.

## 2. Materials and Methods

Six newborn CLP patients, consecutively attending the Orthodontic Department of the University of Brescia Dental School, Brescia, Italy, between July and December 2018, were enrolled in a pilot study comparing digital with non-digital workflow-based PSOT: one operator (D.D.) took patient’s impressions and assigned them to the tray and putty group (three patients) or the digital group (three patients), applying a simple randomization protocol by following the indications of a randomization table; a second operator (L.L.), blind to patient’s group assignments, conducted the PSOT; and two (S.B. and L.V.) different operators, also blind, evaluated the treatment results. The inclusion criteria were as follows: cleft not associated with other malformations, born at term, both parents Caucasian. All subject’s parents gave their informed consent for inclusion before they participated in this pilot study, which was conducted in accordance with the Declaration of Helsinki. The study protocol was approved by the Dental School Scientific Committee of the University of Brescia (2018–079-IOS_CLP).

The first operator visually evaluated impression accuracy immediately after impression realization and repeated the procedure if necessary: an impression was considered of good quality when it was possible to visualize the oral vestibule, alveolar processes, and the hard and soft palate. A T&P impression was evaluated in terms of absence of signs of visible impression material strains or deformations, while an IOS impression was evaluated in terms of absence of scanning holes and splitting into several layers of the reconstruction of a single anatomical surface. The second operator gave an indirect evaluation of impression accuracy, reporting the presence of a bad adaptation in terms of appropriateness of plate extension and absence of compression zones or free spaces between the plate and the intraoral soft tissue, at the time of plate delivery when PSOT started.

Treatment invasiveness was evaluated by means of a questionnaire administered to the patient’s mothers (Table 1). We conducted a literature review to identify if a previously validated questionnaire already existed, without success. We than decided to construct a brief, specific questionnaire based on experts’ judgment, the pertaining literature review and parents’ interviews, using simple and short items assessing single issues [33]. A preliminary pilot test was performed on a group of 20 mothers of orthodontic patients (5–8 years of age) attending for the our Orthodontic Department for the first time, in order to identify any possible source of confusion about any items and, after that, the questionnaire was consequently revised. In the final version of our questionnaire, four answers were available, in order to avoid the possibility of choose the median response as an escape solution in case of doubt, for the following questions: “How much have you been worried after impression taking procedure explanation?”; “Do you think your child suffered during the impression procedure?”; and “How invasive was the procedure compared to what you imagined before impression taking?”. The question “How much have you been worried after impression taking procedure explanation?” was conceived to investigate the psychological effect of the procedure explanation on patient’s mothers, looking at a possible need for improvements in communication techniques, especially when a significant discrepancy is found between the listener’s perception and his opinion after the procedure. The available answers for this question were: “Not at all” when the mother was absolutely secure regarding the procedure; “Slightly” when she was a little concerned, but still assured; “Quite a lot” when she was rather worried; “A lot” when she was very worried and doubtful about the feasibility of the procedure. The question “Do you think your child suffered during the impression procedure?” was conceived to investigate the perception of the mother of her baby’s suffering during the procedure, considering the possibility, for example, of asking the parents to be not present in the room if the impression procedure is a source of further emotional stress for them, or to better prepare them for attending the impression session. The available answers for this question were: “Not at all” when the mother was absolutely secure that her child did not suffer during the procedure; “Slightly” when she thought that her child perceived a little nuisance during the procedure, but no real pain; “Quite a lot” when she thought that her child perceived an important pain during the procedure; “A lot” when she thought that her child perceived an excessive pain during the procedure and she may not be willing to repeat the procedure a second time if needed. The question “Have you found any sign of intra- or extra-oral physical trauma after impression taking?” was conceived to investigate the mother’s perception of physical trauma present after impression-taking, considering not only the actual signs objectively reported from the operators, but also the hypothetical signs perceived as physical trauma by the mother; this could be helpful, for example, when deciding whether or not to spend more time explaining in advance to the mother which signs could the baby might present after the impression procedure, reassuring her of the normality and the mildness of these small traumas. The available answers for this question were: “Yes” when the mother found a sign of intra- or extra-oral physical trauma after impression-taking; “No” when she did not find any sign. The question “How invasive was the procedure compared to what you imagined before impression taking?” was conceived to investigate the perception of the mother of the actual invasiveness of the impression-taking procedure compared to what she supposed based on previous information about the procedure; this information could be helpful in order to better calibrate the information procedure, upon which informed consent is based. The available answers for this question were: “Absolutely less” when the mother was surprised by the ease of the procedure, compared to what she imagined before she attended the impression-taking clinical session; “Slightly less” when she was reassured because the real procedure was a little better compared to what she imagined; “Slightly more” when she was a little disappointed, because the real procedure was a little worse than what she imagined; “Much more” when she was negatively surprised by the invasiveness of the procedure compared to what she imagined before, and she was not sure if she would have authorized the procedure if she had been fully aware of its invasiveness.

In order to directly obtain digital intraoral impressions of the maxilla, the operator used his left-hand thumb and forefinger to retract the lip, cheeks and vestibule, exposing the greatest amount of the surface area of the vestibule; then a second operator separated the upper and the lower jaws using their fore and middle fingers as bite blocks and, with the other hand, cleaned and dried the area to be scanned with a sterile gauze. Considering the dimension of the scanner (CS 3600, Carestream Dental, Atlanta, GA, USA) head compared to the small patient mouth, the IOS itself acted as a bite block during the scanning procedure, which was performed quickly by rotating it from left to right on the palatal side and then sliding it along the edentulous ridge vestibular aspect from the right to the left distobuccal areas [34]. The scanner head was pre-heated, dipping it, while still in the sealed sterile envelope, in 38 °C water; the entire impression procedure took less than thirty seconds.

A similar technique was used with traditional materials. An acrylic tray of appropriate dimensions was chosen by the first operator from a set of perforated mini-trays prepared ad hoc by our lab for this purpose and a third operator prepared the alginate (Millennium, Lascod SpA, Sesto Fiorentino, FI, Italy) and placed it in an appropriate quantity into the tray.

**Figure 1:**
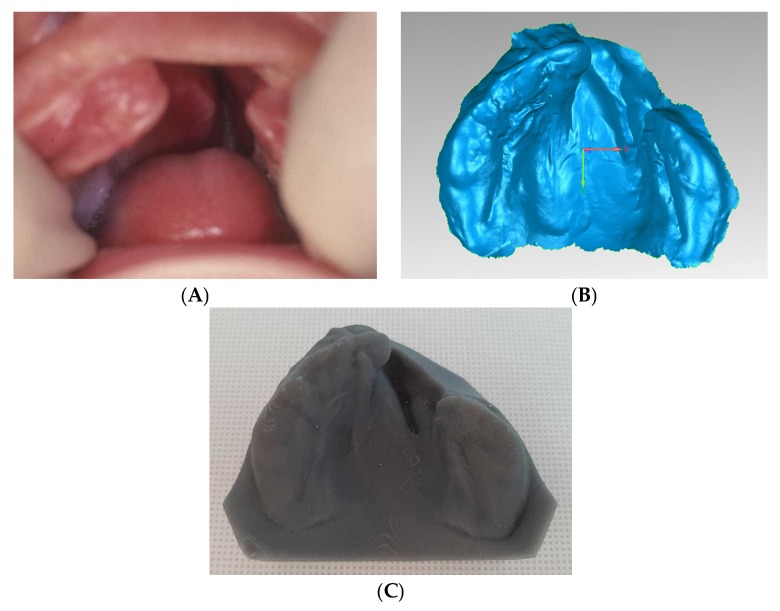
Unilateral cleft lip and palate (UCLP) patient (**A**) clinical picture and corresponding (**B**) digital and (**C**) resin printed models.

**Figure 2:**
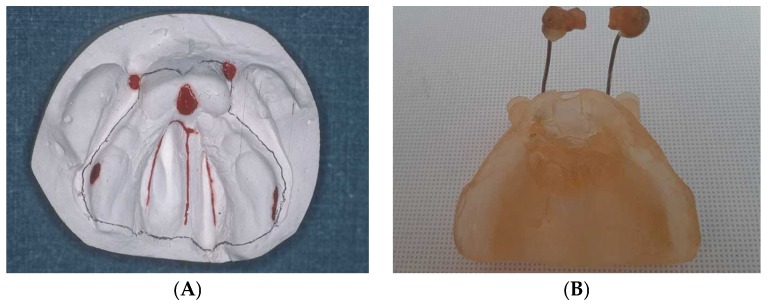
Bilateral cleft lip and palate (BCLP) patient (**A**) stone models and corresponding (**B**) resin plate with two nasal stents.

Virtual impressions were resin printed in order to obtain physical models, and molding plates were fabricated using a conventional acrylic dental resin-based protocol (Figure 1). PSOT with a 24 h worn passive plate, covering the palate and the alveolar ridges and blocking the cleft in both the hard and soft palate, started within the first two weeks after birth. One or two nasal stents, added to the molding plate labial vestibular flange, were used to achieve nasal symmetry and nasal tip projection (Figure 2). The orthopedic appliances were held in place with surgical tapes or with a head bonnet with elastic strips, or with both methods. Patients returned to the clinic every three weeks for plate adjustment and, if necessary, substitution. All patients were administered three to five months of orthopedic treatment before primary gingivoperiosteoplasty. Molding plate was not used after surgery.

**Figure 3:**
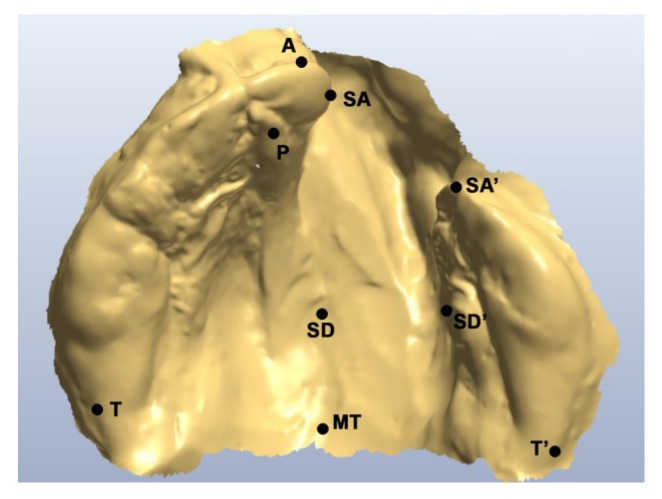
Selected landmarks.

Two operators independently evaluated treatment quality, comparing pre- and post-molding therapy models and extra oral pictures of digital and non-digital protocols patients. The following landmarks (Figure 3) were selected on both pre- and post-molding therapy models: the most anterior point on the greater segment (A), the center of the papilla incisiva (P), the points that have the shortest connection line passing over the anterior cleft (SA and SA’), the tuberal areas (T and T’), the dissecting point on the connecting line between tuberal areas (MT), the points that have the shortest distance passing over the cleft posterior on the hard palate (SD and SD’) (Figure 3). Then, they measured A-MT, SA-SA’, T-T’, and SD-SD’ segments’ length and calculated the difference between pre- and post-molding therapy models, obtaining the outcome measures for statistical analysis [35]. Nasal symmetry and nasal tip projection were evaluated postoperatively. Nasal symmetry was evaluated on frontal cropped pictures on a 3-point ordinal scale in terms of symmetry of alar bases, and the vertical and horizontal dimensions of nostrils: Excellent—symmetry of all three components; Mild asymmetry—asymmetry of one component; Unsatisfactory—asymmetry of more than one component [36]. Nasal tip projection was evaluated on lateral cropped pictures on a 5-point (Excellent, Very good, Good, Satisfactory, Poor) ordinal scale in comparison to standardized reference photographs of treated patients [37]. Surgical records were analyzed to evaluate surgical challenge in terms of time taken for the surgical procedure, extension of subperiosteal dissection, and soft tissue tension after repair: for these last two parameters, the surgeon was asked to express, based on his personal experience and feelings, a judgement of less, equal, or more than usual. 

Descriptive statistics of the maxillary impressions measurements and questionnaire results were calculated, and Mann–Whitney U-test was used to detect the presence of significant differences between IOS and T&P groups both before and after PSOT treatment, whilst the Wilcoxon test was applied for the dependent samples pre- and post-treatment results alone. 

The level of significance was set at *p* < 0.05. SPSS^®^ Statistics software version 20 (IBM Corporation, Armonk, NY, USA) was used for the statistical analysis.

## 3. Results

One patient with bilateral cleft lip and palate (BCLP) and two patients with unilateral cleft lip and palate (UCLP) were enrolled in each group.

### 3.1. Invasiveness and Adverse Events

The following adverse events during impressions-taking were considered: impression material inhalation, impression material ingestion and patient regurgitation.

Adverse events reported during the impression-taking process and number of impression attempts before obtaining a good impression were significantly lower in the digital workflow test group than in the classic impression method control group. In two T&P patients the impression was repeated because some important anatomical details were missing, in one case due to patient regurgitation during the first attempt. No IOS impression was repeated; minor refinements during images acquisition were considered an integral part of a standard IOS impression process and only a complete impression discard was considered as a repetition. Any adverse event was reported in the IOS group. In summary, five impressions were taken in order to have three good quality impressions in the T&P group, while three impressions were enough to obtain three good quality impressions in the IOS group.

Questionnaires results analysis showed that the six mothers of the involved patients considered IOS impressions to be less invasive compared to T&P impressions (Table 2). The clinician that took all the impressions considered the IOS method to be less stressful compared to T&P method.

### 3.2. Plate Manufacturing Accuracy

There were no significant differences between plate manufacturing accuracy based on the virtual digital models face compared to classical stone models: at the time of PSOT commencement, one plate in the T&P group needed a moderate resin grinding in order to avoid palatal tissue compression at the cleft edges level, while one plate in the IOS group needed a shortening at the level of the anterior area of the greater segment; no free spaces between the plate and the intraoral soft tissue were present in either group.

### 3.3. Orthopedic Correction

There were no significant differences between these two protocols in pre-surgical alveolar gap reduction and alveolar cleft segments’ alignment (Table 3). The entire length, measured from point A to MT, remained nearly unchanged (*p* > 0.05), as it occurred to the posterior transversal cleft dimension, measured from point SD to SD’. The cleft width, described by the distance between SA and SA’, had a significant (*p* < 0.05) reduction after PSOT treatment. The posterior dimensions, measured on the basis of the points T and T’, increased (*p* < 0.05) during the treatment period.

**Figure 4:**
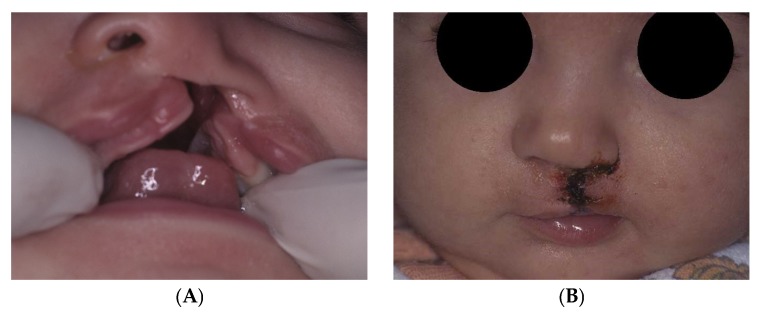
UCLP patient shown in Figure 1 during (**A**) the final stages of PSOT and (**B**) after upper lip surgery.

There were no significant differences in nasal symmetry, nasal tip projection and surgical challenge, evaluated in terms of the time taken for the surgical procedure, extension of subperiosteal dissection, and soft tissue tension after repair (Figure 4). In both groups, one patient received a “Mild asymmetry” and two received an “Excellent” judgement regarding nasal symmetry evaluation. Considering nasal tip projection, one patient received a “Satisfactory”, one a “Good”, and one a “Very good” judgement in both groups. The time taken for the surgical procedure was statistically but not clinically significantly different, with a mean inter-groups difference of 17 ± 5 min. Based on his personal experience and feelings, the surgeon reported standard extension of subperiosteal dissection and soft tissue tension after repair in all six patients.

## 4. Discussion

Pre-surgical orthopedics before primary gingivoperiosteoplasty in newborn CLP patients was proposed for the first time in 1950 and, during the following years, was enthusiastically applied by several clinicians all over the world. Its efficacy was questioned in 1996, following a three-center prospective randomized clinical trial (the DUTCHCLEFT study) that showed that orthopedic treatment did not achieve proposed benefits like growth guidance, development of palatal segments, improved feeding, normalization of tongue posture and minimization of treatment at a later age [38].

Recently, this protocol was improved, adding nasoalveolar molding to previous orthopedic treatment objectives, which improved long-term nasal esthetics, and reduced the number of nasal surgical procedures and the need for secondary alveolar bone grafts, reduced growth disturbance and saved cost through a reduction in the number of surgical hospital admissions [39].

That is why we decided to concentrate on PSOT, looking to ameliorate this technique by means of a reduction in invasiveness and overall cost, maintaining, at the same time, the high clinical standards of the classic technique based on tray and putty impressions and stone models.

The so called “digital revolution” that affected dentistry over the last ten years now involves almost all the different dental specialties, making a growing number of manual tasks easier and faster to perform, cheaper, and more predictable [16]. It started with dental technicians’ labs following the development of different systems of subtractive machining technology, which are now progressively integrated and sometimes substituted by additive processes for layered fabrication, and then included dental therapists, thanks to the diffusion of IOS and CAD-CAM systems designed to realize a chair-side restorative management of all individualized dental devices [40,41,42]. All patients, especially those with special needs, can benefit from a partially or completely digital approach to both diagnostic and treatment processes [43,44,45].

This revolution also involved CLP patients’ treatment in several forms, such as the 3D digital reconstruction of the patient’s face and dental arches for diagnostic purposes, and the CAD/CAM approach to molding plates’ manufacturing process [46,47]. One of the advantages of these digital protocols is the possibility of digitally simulating alveolar segments’ molding and prepare in advance a set of consecutive plates that theoretically do not need any adjustment during the treatment [48,49].

Dealing with cost analysis, several factors must be taken into account, including direct and indirect economical costs and also biological costs, in terms of patients’ suffering [50,51,52]. The cost of a medium quality IOS, ranging from 15000 to 30000 EUR, is surely not comparable with the pure cost of classical impression materials and trays used during a T&P protocol, but, considering that the use of IOS diffusion is rapidly increasing because they can be used in several fields, the cost-per-single use is gradually decreasing, in direct proportion with use. Furthermore, digitally simulating progressive changes in cleft anatomy make it possible to prepare the entire set of molding plates in advance, reducing the need for frequent visits for plate adjustments [53,54]. Nevertheless, the intraoral scanning process, with no risk of impression material inhalation, can be performed without patient transportation to the dental clinic, if a portable IOS is used. Last but not least, from a biological point of view, the reduced invasiveness of this impression technique, demonstrated by the results of the present study, is important, in order to alleviate as much as possible the physical and psychological sufferings of these patients, who are usually orthodontically, orthopedically and surgically treated in several steps until adulthood [55,56,57,58,59].

A comparison from a clinical point of view revealed no differences in clinical efficacy, even if the T&P method also obtained information regarding the initial edges of cleft segments’ nasal side, which could have been used to improve plate stability by inserting a softer material in these undercuts. Nevertheless, the use of extra-oral stabilizing devices, like surgical tapes and head bonnets with elastic strips, was enough to assure that the intraoral plate was held in place, making the use of these undercuts, that sometimes traumatize nasal mucosal tissues and obstruct free space that allows the growth of nasal processes growth and palate cleft reduction, unnecessary.

A limit of this pilot study is the small sample size, with only three patients for each group. This was a consequence of our decision to use this study in to evaluate the feasibility of a future main study by assessing the inclusion and exclusion criteria of the participants, training needs of involved researchers and the expected patients’ recruitment rate [60]. We considered as reasonable a recruitment period of six months, followed by another six months for the treatments’ completion and data analysis, and we decided to enroll CLP patients consecutively attending our hospital, which are usually treats between ten and fifteen newborn CLP patients each year. It will be possible to overcome this limit by organizing a multicenter study, in order to obtain an adequate sample size in a reasonable span of time.

Another limit that could be considered is the plate accuracy evaluation, made only through a clinical evaluation: a different study protocol, repeating both types of impressions on each patient, would have allowed a direct intra-patients comparison of impression and plate accuracy, utilizing more sophisticated tools such as digital superimpositions and colorimetric maps of differences. This is another reason why we consider the present study as a pilot feasibility study.

The same type of limitation could be seen in the absence of a validated questionnaire for the evaluation of mothers’ opinions regarding impression-taking procedure invasiveness. When no existing questionnaires are available, it is appropriate to write a new questionnaire, which should be validated as demonstrating adequate reliability and validity in a representative sample. The questionnaire that we used in this study has been preceded by a careful literature review; it was based on experts’ judgment and parents’ interviews and it was revised after a preliminary pilot test performed on a group of mothers of orthodontic patients, in order to identify any possible source of confusion about any items, but it was not statistically validated in a significant sample of intended respondents.

Nevertheless, our preliminary data indicate that this digital protocol can accelerate the production process of the passive molding plate with an instantaneous transmission of digital impressions to the dental lab, maintaining the same accuracy level and clinical outcomes of classical techniques and reducing the invasiveness of impression taking, avoiding any risk of impression material ingestion or inhalation.

Further applications of this digital approach could also have research relevance, considering, for example, that these digital impressions can be easily repeated during patient growth, and compared between ages with dedicated software for image superimpositions, allowing the study of treatment effects and efficacy on patient growth guidance.

## 5. Conclusions

The results of this pilot study demonstrate that this digital protocol reduces the time, cost and invasiveness of impression-taking. Furthermore, digital impressions are instantaneously available to the dental lab for orthopedic plate design and manufacturing processes.

## Figures and Tables

**Table 1:** Questionnaire administered to patient’s mothers after impression taking in order to evaluate its invasiveness.

Question	Response
How much have you been worried after impression taking procedure explanation?	-Not at all; -Slightly; -Quite a lot; -A lot.
Do you think your child suffered during the impression procedure?	-Not at all; -Slightly; -Quite a lot; -A lot.
Have you found any sign of intra- or extra-oral physical trauma after impression taking?	-Yes;-No.
How invasive was the procedure compared to what you imagined before impression taking?	-Absolutely less; -Slightly less; -Slightly more; -Much more.

**Table 2:** Results of the questionnaire administered to patient’s mothers after impression-taking in order to evaluate its invasiveness, comparing tray and putty (T&P) and intra-oral scanners (IOS) groups. * *p* < 0.05.

Question	Response	T & P n (%)	IOS n (%)
How much have you been worried after impression taking procedure explanation?	-Not at all; -Slightly; -Quite a lot; -A lot.	0 (0%) 1 (33%) 2 (67%) 0 (0%)	2 (67%) 1 (33%) 0 (0%) 0 (0%)
Do you think your child suffered during the impression procedure?	-Not at all; -Slightly; -Quite a lot; -A lot.	0 (0%) 0 (0%) 2 (67%) 1 (33%)	2 (67%) 1 (33%) 0 (0%) 0 (0%)
Have you found any sign of intra- or extra-oral physical trauma after impression taking?	-Yes; -No.	1 (33%) 2 (67%)	0 (0%) 3 (0%)
How invasive was the procedure compared to what you imagined before impression taking?	-Absolutely less; -Slightly less; -Slightly more; -Much more.	0 (0%) 0 (0%) 2 (67%) 1 (33%)	1 (33%) 2 (67%) 0 (0%)

**Table 3:** Differences (expressed in mm) between pre and post pre-surgical orthopedic treatment (PSOT) measurements in traditional vs. digital impression systems. * *p* < 0.05.

Distances	Digital Protocol Differences Pre-Post	T&P Protocol Differences Pre-Post	Digital vs. T&P Protocol Mean Differences
A-MT	−0.6 ± 1.5	−0.4 ± 1.3	−0.2 ± 1.6
SA-SA’	−4.6 ± 2.8	−4.9 ± 3.1	−0.3 ± 3.3
SD-SD’	−0.8 ± 1.1	−1.1 ± 0.8	−0.3 ± 1.2
T-T’	2.9 ± 1.9	2.6 ± 1.2	−0.2 ± 12.1

A-MT = distance between the most anterior point on the greater segment and the dissecting point on the connecting line between tuberal areas. SA − SA’ = distance between the points, on the greater and the smaller segment, that have the shortest connection line passing over the anterior cleft. SD − SD’ = distance between the points, on the greater and the smaller segment, that have the shortest distance passing over the clef posterior on the hard palate. T − T’ = distance between the tuberal areas. T&P = Tray and Putty.
